# Risk factors for distal junctional kyphosis after posterior spinal surgery in adolescent idiopathic scoliosis: a meta-analysis

**DOI:** 10.3389/fsurg.2023.1263655

**Published:** 2023-10-18

**Authors:** Ruili Jia, Yubin Long

**Affiliations:** ^1^ Department of Nephrology, The First Central Hospital of Baoding, Baoding, China; ^2^ Department of Orthopedics, The First Central Hospital of Baoding, Baoding, China

**Keywords:** risk factors, distal junctional kyphosis, posterior spinal surgery, adolescent idiopathic scoliosis, meta-analysis

## Abstract

**Introduction:**

Distal junctional kyphosis (DJK) is a serious complication after posterior spinal surgery in managing adolescent idiopathic scoliosis (AIS). Our study aims to investigate the predictors of DJK in AIS patients.

**Methods:**

We searched the English databases of PubMed, Embase, and the Cochrane Library for clinical research studies on AIS. To identify the risk factors for DJK, we collected demographic data, such as age, gender, and body mass index (BMI), and sagittal parameters, including preoperative pelvic tilt (PT), sagittal vertical axis (SVA), lumbar lordosis (LL), thoracic kyphosis (TK), thoracolumbar kyphosis (TLK), distal junctional angle (DJA), lowest instrumented vertebrae (LIV) relative to sagittal stable vertebrae (SSV), and postoperative DJA. Data were analyzed by RevMan (5.3 version) and STATA (12.0 version).

**Results:**

Finally, six included articles (1,240 patients) showed 9% (98 of 1,240 patients) of patients suffering from DJK. Our findings indicated that preoperative TK [*p* = 0.007, OR = 0.35, 95% CI (0.10, 0.61)], TLK [*p* < 0.0001, OR = 5.99, 95% CI (3.33, 8.65)], and postoperative DJA [*p* = 0.002, OR = 0.56, 95% CI (0.21, 0.91)] in the DJK group were markedly higher than those in the non-DJK group. Moreover, patients with LIV above SSV [*p* < 0.0001, OR = 7.95, 95% CI (4.16, 15.22)] were more likely to develop DJK. No discernible difference was found in age, sex, BMI, preoperative PT, SVA, LL, or DJA between the two groups.

**Conclusions:**

Regarding the AIS patients, factors such as higher preoperative TK and TLK, higher postoperative DJA, and LIV above the SSV were related to an increased rate of DJK.

## Introduction

Adolescent idiopathic scoliosis (AIS), defined as a Cobb angle of at least 10°, affects 1%–3% of adolescents (1, 2). When the Cobb angle of the primary curve is greater than 45° together with exacerbated symptoms and functional capabilities, surgery is the optimal option (3, 4). Long posterior instrumentation has been widely applied in treating AIS because it can provide planar correction in three planes and stable fixation (5–8). However, distal junctional kyphosis (DJK), defined as either focal kyphosis at lowest instrumented vertebrae (LIV)-LIV + 1 >10° or >5° of kyphotic change in the sagittal disc angle between LIV and LIV + 1 after surgery or any follow-up, caused by long fusion inevitably results in imbalance and unacceptable deformity (9). Thus, identifying the predictors related to DJK is an urgent need to prevent DJK. Postoperative thoracic kyphosis (TK) ≥25° and thoracolumbar kyphosis (TLK) ≥10° are related to DJK (10). Segal (11) demonstrated that the LIV chosen proximal to the sagittal stable vertebra (SSV) was a risk factor for DJK.

Many papers have been published to identify risk factors for DJK, yet they are still controversies. It is urgently needed to further clarify risk factors to avoid similar problems. To our knowledge, no meta-analysis has been performed to identify the predictors of DJK in AIS patients. Therefore, we performed a meta-analysis to investigate the predictors of DJK after posterior surgery in treating AIS.

## Methods

### Search strategy

We searched the English databases of PubMed, Embase, and Cochrane Library with the following keywords: “distal junctional kyphosis,” “adolescent idiopathic scoliosis,” and “risk factors.” We searched published studies up to May 2023.

### Eligibility criteria

Inclusion criteria were as follows: (1) adolescent patients and (2) prospective, retrospective, and comparative research studies on risk factors of DJK in treating AIS. Exclusion criteria were as follows: (1) abstracts, letters, reviews, or case reports; (2) repeated data; (3) not including data of interest; (4) patients with tumors, infection, or inflammation; and (5) patients with a history of spinal surgery.

### Data extraction and outcome measures

The information covered the study's overall features and the measured outcomes. To minimize duplication of material, we kept only the most useful article or entire study where the same population was described in many publications. Two authors extracted the data separately. Discussion and consensus were used to settle any differences on paper eligibility. The possibility of publishing bias was checked. A statistician checked the funnel plot for publication bias with a visual assessment. An asymmetric funnel plot indicates publication bias, and a symmetric one means no publication bias. The funnel plot asymmetry was measured using the Egger and Begg tests with a significance threshold of *p* < 0.10. The effect of publication bias was estimated using the trim and fill method. We did not perform sensitivity analysis due to the low heterogeneity of each component.

### Definition of sagittal parameters

*LIV + 1*: the vertebra just distal to the LIV;

*DJK*: either focal kyphosis at LIV-LIV + 1 >10° or >5° of kyphotic change in the sagittal disc angle between LIV and LIV + 1 after surgery or any follow-up;

*Distal junctional angle (DJA)*: sagittal disc angle between LIV and LIV + 1;

*Stable sagittal vertebrae (SSV)*: defined as the vertebral level at which 50% of the vertebral body was in front of the posterior sacral vertical line on a standing lateral radiograph;

*Thoracic kyphosis (TK)*: Cobb angle between the upper endplate of T5 and the lower endplate of T12;

*Thoracolumbar kyphosis (TLK)*: Cobb angle between the upper endplate of T11 and the lower endplate of L2;

*Lumbar lordosis (LL)*: Cobb angle between the lower endplate of T12 and the upper endplate of S1;

*Pelvic tilt (PT)*: angle between the vertical line and the line joining the hip axis to the center of the superior endplate of S1;

Sagittal vertical axis (SVA): the horizontal distance between the posterior corner of the sacrum and the C7 plumb line.

### Statistical analysis

We estimated odd ratios (ORs) and 95% confidence intervals (CIs) for the data. A *p*-value of 0.05 was used to indicate statistical significance. Depending on the heterogeneity of the papers considered, random-effects or fixed-effects models were utilized. The chi-squared test and the *I*-squared test were used to evaluate heterogeneity, with a *p*-value of 0.10 for the chi-squared test and *I*^2 ^> 50% implying heterogeneity. Review Manager version 5.3 (The Cochrane Collaboration, Oxford, UK) and STATA 12.0 (Stata Corporation, College Station, TX, USA) were used for all statistical analyses.

## Results

### Study identification and selection

**Figure 1:**
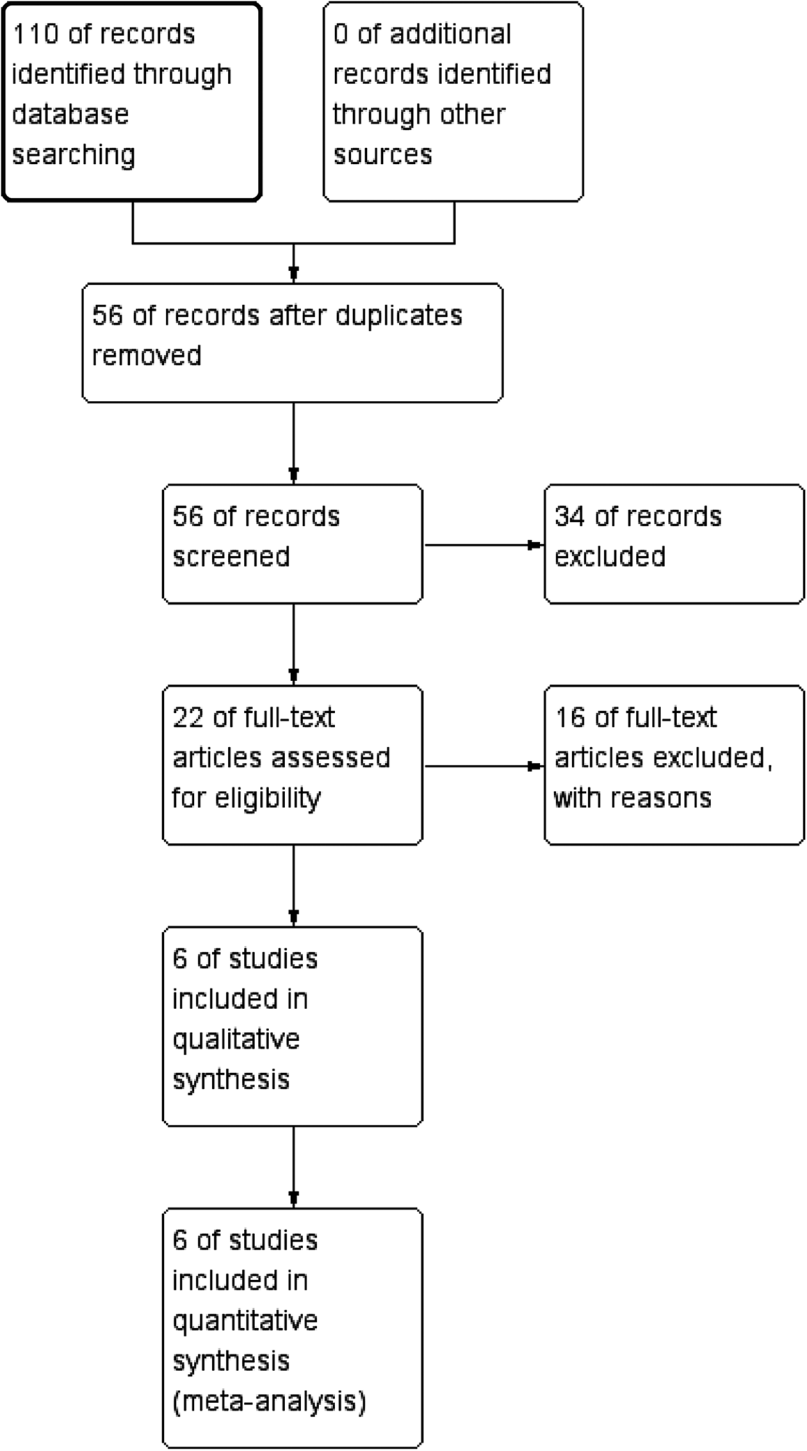
Flow diagram of study selection.

Initially, the database search found 110 English publications. Due to repetition, 54 papers were excluded, and 34 articles were removed after reviewing according to titles and abstracts. The remaining 22 articles were retrieved. Thirteen papers were eliminated because they did not focus on adolescents, and three articles were removed since they did not show data of our interest. Finally, the meta-analysis included six articles that matched our inclusion criteria. The selection procedure used in this meta-analysis is depicted in Figure 1.

### Baseline characteristics and quality assessment

Table 1 shows the key features of the six publications (1,240 patients) included in the meta-analysis published before May 2023.

**Table 1:** Characteristics of included studies.

First author	Year	Country	No. of participants		Study type
			DJK	Total	
Wang (10)	2021	China	8	42	Respective
Segal (11)	2020	USA	23	346	Respective
Ameri (12)	2011	Iran	9	130	Respective
Segal (13)	2021	USA	29	498	Respective
Marciano (14)	2021	USA	21	111	Respective
Yang (15)	2018	USA	8	113	Respective

We utilized the Newcastle–Ottawa quality assessment scale (NOQAS) to assess the quality of each study because they were all retrospective. This scale was used to assign a maximum of nine points for the quality of selection, comparability, exposure, and outcomes for research participants in nonrandomized case–control studies and cohort studies. Four studies received eight points, while the other two received seven. As a result, the quality of the included studies was good (Table 2).

**Table 2:** Quality assessment according to the Newcastle–Ottawa quality assessment scale (NOQAS) of each study.

Study	Selection	Comparability	Exposure	Total score
Wang (10)	3	3	2	8
Segal (11)	3	3	2	8
Ameri (12)	3	2	3	8
Segal (13)	2	3	2	7
Marciano (14)	3	2	3	8
Yang (15)	3	2	2	7

### Age

**Figure 2:**
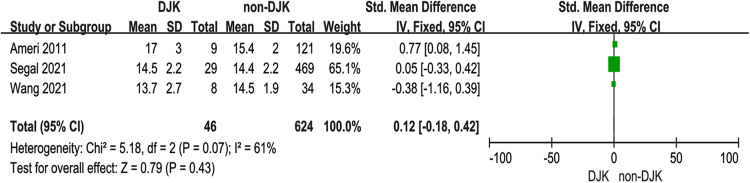
Forest plot showing age in two groups. CI, confidence interval; df, degree of freedom; M–H, Mantel–Haenszel.

Three studies (10, 12, 13) investigated whether age affects DJK. The studies had high heterogeneity (*p* for heterogeneity = 0.07, *I*^2 ^= 61%, Figure 2). The result showed that age at the time of surgery was not associated with DJK [fixed-effects model; *p* = 0.43, OR = −0.12, 95% CI (−0.18, 0.42), Figure 2].

### Sex

**Figure 3:**
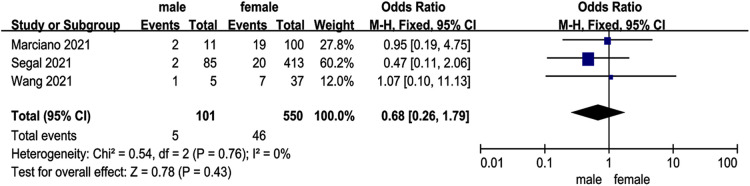
Forest plot showing sex in two groups. CI, confidence interval; df, degree of freedom; M–H, Mantel–Haenszel.

Three studies (10, 13, 14) investigated the relationship between sex and DJK. The studies had low heterogeneity (*p* for heterogeneity = 0.76, *I*^2 ^= 0%, Figure 3). The result showed that sex was not associated with DJK [fixed-effects model; *p* = 0.43, OR = 0.68, 95% CI (0.26, 1.79), Figure 3].

### Body mass index

**Figure 4:**
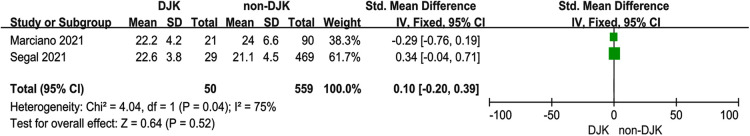
Forest plot showing body mass index in two groups. CI, confidence interval; df, degree of freedom; M–H, Mantel–Haenszel.

Two studies (13, 14) investigated whether body mass index (BMI) affects DJK. The studies had high heterogeneity (*p* for heterogeneity = 0.04, *I*^2 ^= 75%, Figure 4). The result showed that BMI was not associated with DJK [fixed-effects model; *p* = 0.52, OR = 0.10, 95% CI (−0.20, 0.39), Figure 4].

### Preoperative pelvic tilt

**Figure 5:**
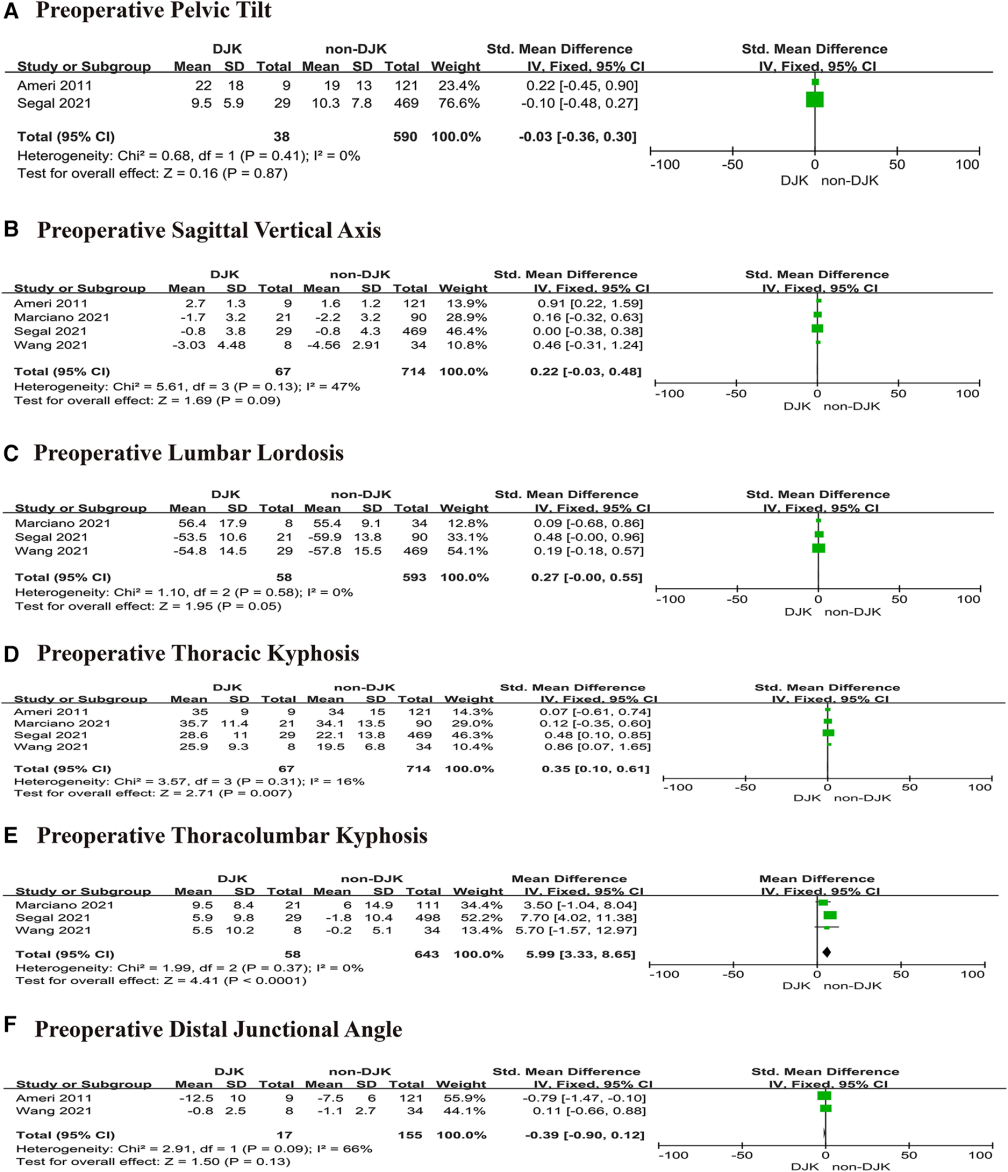
Forest plot showing preoperative sagittal parameters. (**A**) Forest plot showing pelvic tilt in two groups; (**B**) forest plot showing sagittal vertical axis in two groups; (**C**) forest plot showing lumbar lordosis in two groups; (**D**) forest plot showing thoracic kyphosis in two groups; (**E**) forest plot showing thoracolumbar kyphosis in two groups; and (**F**) forest plot showing the preoperative distal junctional kyphosis in two groups. CI, confidence interval; df, degree of freedom; M–H, Mantel–Haenszel.

Two studies (12, 13) investigated the relationship between preoperative PT and DJK. The studies had low heterogeneity (*p* for heterogeneity = 0.41, *I*^2 ^= 0%, Figure 5A). The result showed that PT was not associated with DJK [fixed-effects model; *p* = 0.87, OR = −0.03, 95% CI (−0.36, 0.30), Figure 5A].

### Preoperative sagittal vertical axis

Four studies (10, 12–14) investigated the relationship between preoperative SVA and DJK. There was no significance in the test for heterogeneity, thus the studies had low heterogeneity (*p* for heterogeneity = 0.13, *I*^2 ^= 47%, Figure 5B). The result showed that SVA was not associated with DJK [fixed-effects model; *p* = 0.09, OR = 0.22, 95% CI (−0.03, 0.48), Figure 5B].

### Preoperative lumbar lordosis

Three studies (10, 13, 14) investigated the relationship between preoperative LL and DJK. The studies had low heterogeneity (*p* for heterogeneity = 0.58, *I*^2 ^= 0%, Figure 5C). The result showed that LL was not associated with DJK [fixed-effects model; *p* = 0.05, OR = 0.27, 95% CI (−0.00, 0.55), Figure 5C].

### Preoperative thoracic kyphosis

Four studies (10, 12–14) investigated the relationship between preoperative TK and DJK. The studies had low heterogeneity (*p* for heterogeneity = 0.31, *I*^2 ^= 16%, Figure 5D). The result showed that TK was a risk factor for DJK [fixed-effects model; *p* = 0.007, OR = 0.35, 95% CI (0.10, 0.61), Figure 5D].

### Preoperative thoracolumbar kyphosis

Three studies (10, 13, 14) investigated the relationship between preoperative TLK and DJK. The studies had low heterogeneity (*p* for heterogeneity = 0.37, *I*^2 ^= 0%, Figure 5E). The result showed that TLK was a risk factor for DJK [fixed-effects model; *p* < 0.0001, OR = 5.99, 95% CI (3.33, 8.65), Figure 5E].

### Preoperative distal junctional angle

Two studies (10, 12) investigated the relationship between preoperative DJA and DJK. The studies had high heterogeneity (*p* for heterogeneity = 0.09, *I*^2 ^= 66%, Figure 5F). The result showed that DJA was not associated with DJK [fixed-effects model; *p* = 0.13, OR = −0.39, 95% CI (−0.90, 0.12), Figure 5F].

### Lowest instrumented vertebrae relative to sagittal stable vertebrae

**Figure 6:**
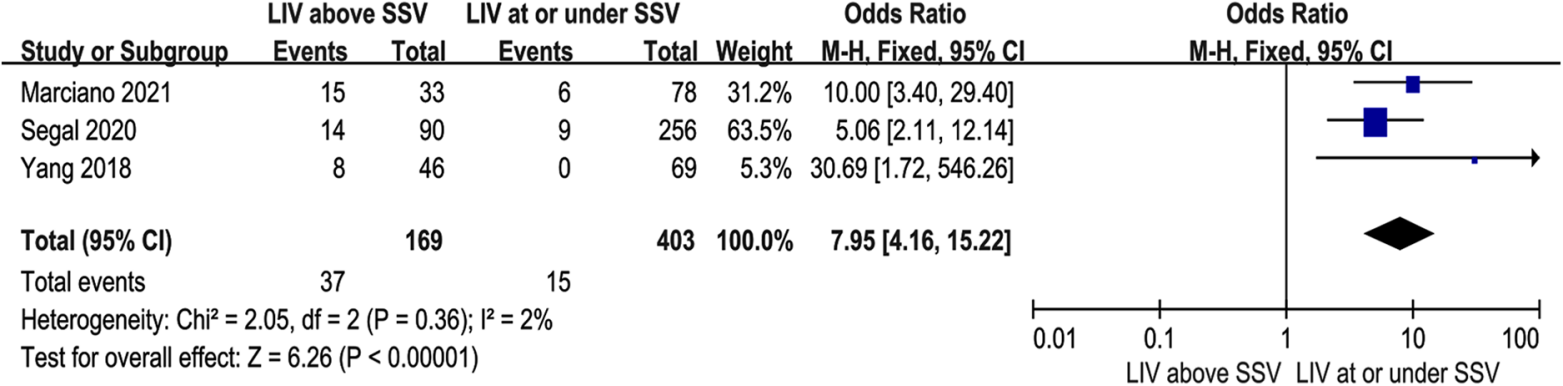
Forest plot showing the lowest instrumented vertebrae relative to sagittal stable vertebrae in two groups. CI, confidence interval; df, degree of freedom; M–H, Mantel–Haenszel.

Three studies (11, 14, 15) investigated whether LIV relative to SSV affected DJK. There was no significance in the test for heterogeneity; thus, the studies had low heterogeneity (*p* for heterogeneity = 0.36, *I*^2 ^= 2%, Figure 6). The result showed that postoperative LIV above SSV was a risk factor for DJK [fixed-effects model; *p* < 0.0001, OR = 7.95, 95% CI (4.16, 15.22), Figure 6].

### Postoperative distal junctional angle

**Figure 7:**
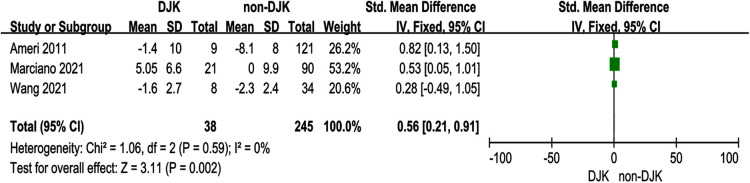
Forest plot showing the postoperative distal junctional kyphosis in two groups. CI, confidence interval; df, degree of freedom; M–H, Mantel–Haenszel.

Three studies (10, 12, 14) investigated the relationship between postoperative DJA and DJK. There was no significance in the test for heterogeneity; thus, the studies had low heterogeneity (*p* for heterogeneity = 0.59, *I*^2 ^= 0%, Figure 7). The result showed that postoperative DJA was a risk factor for DJK [fixed-effects model; *p* = 0.002, OR = 0.56, 95% CI (0.21, 0.91), Figure 7].

### Publication bias

No publication bias was found for all included studies (all *p *> 0.05) after detection by STATA 12.0.

## Discussion

As a serious consequence of posterior spinal surgery, DJK causes local pain, imbalance, poor cosmesis, and increases mechanical stress on neighboring levels, contributing to adjacent segment degeneration (16–20). It is important to identify the risk factors to assist surgeons in lowering the rate of DJK. To our knowledge, no meta-analysis has been reported on the topic. Thus, we performed a meta-analysis to investigate the predictors of DJK after posterior surgery in treating AIS. To our knowledge, this is the first meta-analysis on this topic. In the present study, the incidence of DJK was 7.9% (98 of 1,240 patients), and our findings showed that preoperative TK and TLK and postoperative DJA in the DJK group were markedly higher than in the non-DJK group. Moreover, patients with LIV above the SSV were associated with the development of DJK. However, no obvious difference was found in age, sex, BMI, preoperative PT, SVA, LL, or DJA between the two groups.

Until now, the selection of LIV in patients with AIS receiving thoracic fusion remains controversial. Pain, disc degeneration, and mobility difficulties have all been linked to posterior spinal fusion, with instrumentation reaching the lower lumbar spine (21–24). Choosing a proximal LIV can retain motion, but it may increase the risk of developing an imbalance below this level in the coronal or sagittal plane, such as DJK. Based on recent publications (9), the rate of DJK ranged from 0.2% to 15%. According to the study of Yang (15), 17% of AIS patients had DJK if the LIV was above SSV but no DJK if the LIV was not above SSV. The result was similar to Segal's research (11). In the present study, the rate of DJK was 13.2% (36 of 273 patients) in the LIV above SSV group and 0.8% (4 of 472 patients) in the LIV under SSV group, implying that LIV above SSV was closely associated with DJK. It is important to note that terminating fusion at the thoracolumbar junction increases the risk of DJK development. Spinal surgeons should debate and investigate the decision to use LIV to preserve spinal mobility while reducing the risk of distal junctional disorders.

It is well known that preoperative TK and TLK are two crucial factors that determine the success or failure of the surgery and the incidence of complications like pain and DJK when surgeons establish surgical plans. Segal (13) demonstrated that TK and TLK were significantly larger in the DJK group than in the non-DJK group. However, Wang (10) and Marciano (14) discovered that preoperative TK and TLK were not related to DJK. However, Segal (13) demonstrated that TK and TLK were significantly higher in the DJK group than in the non-DJK group. In our study, both high preoperative TK and TLK were associated with the development of DJK, implying that a more severe sagittal deformity before surgery might be a risk for DJK. Despite the fact that Wang (10) observed no evident differences among preoperative TK and TLK and DJK, he indicated that postoperative TK >25° or postoperative TLK >10° were related to DJK. Due to a lack of papers, we could not get data for postoperative TK and TLK. More studies on this topic will be required in the future.

There was considerable debate on whether perioperative DJA affected the rate of DJK. Wang (10) revealed that DJA was not a risk factor for DJK after surgery. Conversely, Marciano (14) claimed that postoperative DJA was linked to an increased incidence of DJK. We also assessed whether DJA was correlated with the development of DJK before and after surgery. Preoperative DJA did not differ significantly between the two groups, but postoperative DJA was significantly higher in the DJK group than in the non-DJK group, suggesting that high postoperative DJA was a critical risk factor for DJK. Compared with <5° DJA, >5° DJA raised the rate of developing DJK by nearly 16-fold (10). Nonetheless, we should be aware of the substantial heterogeneity (*I*^2 ^= 66%) in preoperative DJA and the low heterogeneity (*I*^2 ^= 0%) in postoperative DJA.

Although we provided some novel insights, there were still several limitations. First, we did not find an randomized controlled trial (RCT) article related to this topic, which is needed in the further study. Second, due to the small number of included studies, some sagittal parameters, such as pelvic incidence and T1 pelvic angle, could not be analyzed. In addition, we could not perform subgroups of AIS due to a lack of included studies. Third, we only searched for English articles; thus, we did not include articles published in other languages due to difficulties in obtaining accurate medical translations. However, to our knowledge, this is the first meta-analysis to investigate the predictors of DJK after posterior surgery in treating AIS.

In summary, many predictors, especially sagittal parameters, including higher preoperative TK and TLK, higher postoperative DJA, and LIV above the SSV, were risk factors for DJK in AIS patients after posterior surgery. When faced with AIS patients who require surgery, we expect our findings to help the surgeon decide which techniques to use. Future research should incorporate more studies.
